# Simultaneous Detection of Dihydroxybenzene Isomers Using Electrochemically Reduced Graphene Oxide-Carboxylated Carbon Nanotubes/Gold Nanoparticles Nanocomposite

**DOI:** 10.3390/bios11090321

**Published:** 2021-09-07

**Authors:** Angélica Domínguez-Aragón, Rocio B. Dominguez, Erasto Armando Zaragoza-Contreras

**Affiliations:** 1 Centro de Investigación en Materiales Avanzados, S.C., Miguel de Cervantes No. 120, Chihuahua C.P. 31136, Chih, Mexico; angelica.dominguez@cimav.edu.mx; 2 CONACyT-Centro de Investigación en Materiales Avanzados, S.C., Miguel de Cervantes 120, Chihuahua C.P. 31136, Chih, Mexico; berenice.dominguez@cimav.edu.mx

**Keywords:** dihydroxybenzene isomers, simultaneous detection, carboxylated carbon nanotubes, electrochemically reduced graphene oxide, nanocomposite, hydroquinone, catechol, resorcinol

## Abstract

An electrochemical sensor based on electrochemically reduced graphene oxide (ErGO), carboxylated carbon nanotubes (cMWCNT), and gold nanoparticles (AuNPs) (GCE/ErGO-cMWCNT/AuNPs) was developed for the simultaneous detection of dihidroxybenzen isomers (DHB) hydroquinone (HQ), catechol (CC), and resorcinol (RS) using differential pulse voltammetry (DPV). The fabrication and optimization of the system were evaluated with Raman Spectroscopy, SEM, cyclic voltammetry, and DPV. Under optimized conditions, the GCE/ErGO-cMWCNT/AuNPs sensor exhibited a linear concentration range of 1.2–170 μM for HQ and CC, and 2.4–400 μM for RS with a detection limit of 0.39 μM, 0.54 μM, and 0.61 μM, respectively. When evaluated in tap water and skin-lightening cream, DHB multianalyte detection showed an average recovery rate of 107.11% and 102.56%, respectively. The performance was attributed to the synergistic effects of the 3D network formed by the strong π–π stacking interaction between ErGO and cMWCNT, combined with the active catalytic sites of AuNPs. Additionally, the cMWCNT provided improved electrocatalytic properties associated with the carboxyl groups that facilitate the adsorption of the DHB and the greater amount of active edge planes. The proposed GCE/ErGO-cMWCNT/AuNPs sensor showed a great potential for the simultaneous, precise, and easy-to-handle detection of DHB in complex samples with high sensitivity.

## 1. Introduction

The simultaneous detection of dihydroxybenzene isomers (DHB), namely hydroquinone (HQ), catechol (CC), and resorcinol (RS), is of significant concern given their harmful effects on human health. However, due to their great versatility, it is expected that the worldwide production of DHB will continue to grow at a high pace, resulting in high exposure for the population through several pathways such as water and food contamination, environmental pollution, and cosmetic usage. For example, HQ is used in a wide range of industrial activities such as synthesis and formulation of rubbers and plastics, in food, and in cosmetics dedicated to skin lightening due to HQ inhibition over melanin production [1,2]. However, because HQ is a derivative of benzene, the consternation regarding its toxicity and carcinogenic behavior [3,4,5] has encouraged a significant number of investigations. HQ is also linked to depigmentation of the skin (vitiligium) and conditions in eyes, and in the respiratory system due to high exposure [1]. CC is also a DHB that can be used as an ingredient in cosmetics and pharmaceuticals industries [6]. When absorbed in the gastrointestinal tract, CC can produce renal tube destruction, liver function reduction, and strong central nervous system suppression when exposed to high doses [7]. RS is the third DHB, and has been related to angioneurotic edema, eczema, and urticaria [8]. After prolonged exposure to RS, suppression of thyroid hormone synthesis in humans, hematological abnormalities, carcinogenesis, and fatal cases of human fetus poisoning have been observed [9]. Since HQ, CC, and RS are generally used in the production of dyes, cosmetics, pesticides, and some medicines, the DHB often coexist in waste waters and natural waters. Therefore, they are classified as priority toxic pollutants for their high toxicity and the degradation-resistant properties in the environment [10,11]. 

Consequently, to mitigate their harmful effects on human health, it is of great importance to maintain control of DHB dosage in the recommended limits emitted by the different regulatory entities, such as the World Health Organization (WHO) [12]. Thus, the precise and fast detection of DHB is a major concern for the environmental analysis.

Conventional analytical techniques such as chromatography, fluorescence, and spectrometry are the first choice, but electrochemical multianalyte sensors can be a precise and easy-to-handle alternative for a quick and efficient simultaneous determination of DHB. Previously, biosensors based on polyphenol oxidase have been designed for phenol detection, achieving remarkable sensitivity and selectivity [13]. However, drawbacks such as low chemical and thermal stability along with high cost has promoted detection in non-enzymatic fashion [14]. However, due to their similar structure and closeness of oxidation potential, the multidetection of HQ, CC, and RS is not feasible with conventional electrodes such as glassy carbon electrodes (GCE), and surface modification is necessary. Intensive research work for novel chemically modified electrodes (CME) for multianalyte detection includes metal-organic frameworks (MOF) [15,16], conductive polymers [17], metal nanoparticles [18], cobalt-phthalocyanine [19], and carbon-based nanomaterials such as graphene and carbon nanotubes (CNT) [20,21]. 

Among them, hybrids of CNT and graphene have shown high potential for detecting redox molecules [22], given their improved conductivity, large surface area, and catalytic properties compared with either pristine CNTs or GO/graphene [23]. The strong π–π stacking interaction generated by the 3D network, formed by the combination of graphene of high charge density and CNTs of large surface area, enables synergistic effects with enhanced mechanical, optical, electrical, and electrochemical properties [24].

Usually, for ease of synthesis, multi-walled carbon nanotubes (MWCNT) are included instead of single-walled carbon nanotubes (SWCNT), showing porous structure, high surface area, excellent chemical stability, good mechanical properties, and ability to promote electron transfer reactions [25]. However, since MWCNT are insoluble, the processability for surface modification can be difficult. To overcome this, the carboxyl functionalization of MWCNT can enhance its hydrophilicity, making it more dispersible to prepare a uniform electrode layer. Carboxylated MWCNT (cMWCNT) have shown higher attachment properties due to the functional groups on their surface, which help to generate a more homogeneous surface with better electrochemical activity [26]. Additionally, the carboxylation of MWCNT produces an improved electrocatalytic activity in the material compared with unmodified CNT. 

On the other hand, the preparation of CME by electrochemical techniques offers advantages such as environmental safety, simplified synthesis steps, and low reagent consumption. Carbon-based nanocomposites of electrochemically reduced graphene oxide (ErGO) and MWCNT can be prepared within a single step, avoiding common drawbacks such as agglomeration and restacking of layers. Moreover, the ErGO-MWCNT nanocomposite can be used as an excellent scaffold for catalytic nanomaterials deposition, such as gold nanoparticles (AuNPs) [20]. The AuNPs provide additional features such as excellent electrical properties, large surface-to-volume ratio, and high surface reaction activity, resulting in a high-performance electrode surface [27].

In this work, we propose a CME for simultaneous detection of HQ, CC, and RS using an ErGO-cMWCNT/AuNPs nanocomposite prepared by successive electrochemical steps. Surface characterization revealed a fast electron transfer rate constant, high charge transfer, and excellent analytical response towards the detection of DHB. The proposed sensor was also demonstrated for detection in environmental and cosmetic samples, showing excellent accuracy regarding the HQ, CC, and RS content.

## 2. Materials and Methods

### 2.1. Characterization

Morphology was analyzed with a JEOL 300-S (Tokyo, Japan) scanning electron microscope (SEM), and Raman spectroscopy was analyzed using a microspectrometer (Horiba, LabRAM-HR) (Horiba, Kyoto, Japan). A three-electrode arrangement, including a GCE (ø = 3 mm), a Pt wire, and an Ag/AgCl, were used as the working electrode, counter electrode, and reference electrode, respectively. Cyclic voltammetry (CV), electrochemical impedance spectroscopy (EIS), and differential pulse voltammetry (DPV) techniques were performed using an EmStat3 + blue (PalmSens, Houten, The Netherlands) potentiostat.

### 2.2. Materials and Reagents

HAuCl_4_.3H_2_O, K_4_[Fe(CN)_6_], K_3_[Fe(CN)_6_], HQ, CC, and RS were obtained from Sigma Aldrich (St. Louis, MO, USA). H_2_SO_4_ and HNO_3_ were provided from Fermont (Monterrey, Nuevo León, Mexico). Phosphate buffer solution (PBS) (0.01 M) was prepared from NaCl, KCl, Na_2_HPO_4_, and KH_2_PO_4_ (Sigma-Aldrich, St. Louis, MO, USA). GO was prepared using the Hummers method [28], and the cMWCNT were carboxylated by acid treatment [29]. A skin-lightening cosmetic cream at 4% of HQ (Bustillos Laboratory, Mexico D.F, Mexico) was obtained at a local store.

### 2.3. GCE Modification with ErGO-cMWCNT/AuNPs Nanocomposite

Prior to modification, GCE was polished with alumina suspension (0.05 µm) and rinsed with deionized water, followed by sonication in ethanol and deionized water. Then, 4 µL of GO (5 mg/mL) and cMWCNT (5 mg/mL) (1:1) mixture were drop cast on the GCE surface and allowed to dry. CV was carried out on the GCE/GO–cMWCNT in PBS (0.01 M, pH 7.4) at a potential window from −1.5 to 0.5 V and scan rate of 100 mV s^−1^ during 20 cycles of reduction. The modified electrode GCE/ErGO-cMWCNT was washed with distilled water and dried at room temperature.

The AuNPs were electrodeposited on the nanocomposite to increase the electrochemically active surface. Briefly, the AuNPs were electrodeposited from a 1 mM solution of HAuCl_4_ in 0.1 M H_2_SO_4_ using a constant potential of −0.5 V for 80 s.

**Figure 1:**
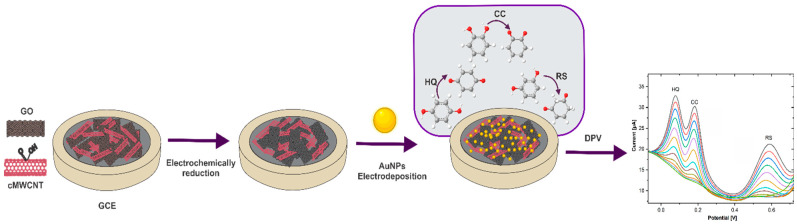
Scheme of the step-by-step preparation of the GCE/ErGO-cMWCNT/AuNPs sensor for simultaneous detection of DHB.

Finally, the GCE/ErGO-cMWCNT/AuNPs was rinsed with distilled water and dried. AuNPs were also electrodeposited on GCE/ErGO, GCE/cMWCNT, and GCE using the same conditions to corroborate synergistic effects of the nanocomposite. Figure 1 illustrates the steps of the GCE modification.

### 2.4. Electrochemical Measurements and Real Sample Analysis

The electrochemical analysis was performed in a cell containing 25 mL of PBS 0.01 M at pH 7.0. Different concentrations of HQ, CC, and RS were measured both separately and simultaneously by DPV. The potential window was from −0.4 to 0.9 V at a scan rate of 0.05 V s^−1^. HQ, CC, and RC were detected simultaneously in tap water diluted in PBS (0.01 M, pH 7) (X10). A skin-lightening cream was diluted in PBS (0.01 M, pH 7) (X10) to obtain three concentrations of HQ. The cream was spiked with CC and RS to corroborate the simultaneous determination of these isomers.

## 3. Results and Discussion

### 3.1. Materials Characterization

#### 3.1.1. SEM

**Figure 2:**
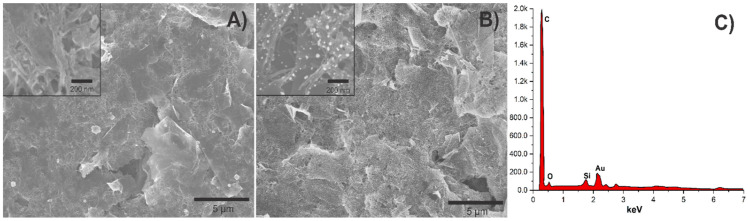
SEM images of (**A**) ErGO-cMWCNT, (**B**) ErGO-cMWCNT/AuNPs, and (**C**) EDS of ErGO-cMWCNT/AuNPs.

The morphology of the ErGO-cMWCNT and ErGO-cMWCNT/AuNPs composites was characterized by SEM. The electrochemical reduction of GO over the GCE produced numerous interconnected layers partially folded and wrinkled, as observed in previous ErGO morphology [30]. ErGO sheets, partially wrinkled, were formed attached parallel to the electrode surface. ErGO-cMWCNT formed an interconnected porous network, increasing the surface roughness, as shown in Figure 2A. The ErGO-cMWCNT also served as a scaffold for growing well-dispersed AuNPs of around 20–30 nm in diameter, as observed in Figure 2B. AuNPs were equally prone to deposit over the basal plane of ErGO sheets and tubular cMWCNT, forming a favorable pathway in the composite for electronic transfer. The EDS analysis confirmed (Figure 2C) the presence of Au element in the nanocomposite.

#### 3.1.2. Raman

**Figure 3:**
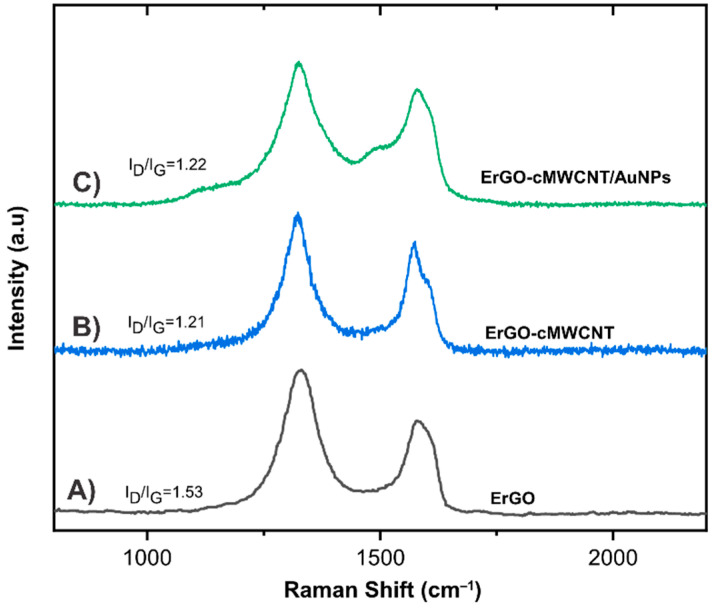
Raman spectra of (**A**) ErGO, (**B**)ErGO-cMWCNT, (**C**) ErGO-cMWCNT/AuNPs.

Raman spectroscopy is important to characterize carbon-based materials such as graphene and MWCNT, since it provides information on structural defects. Figure 3 shows the D band around 1325 cm^−1^, which is related to the degree of defects and disorder in the graphene structure, and the G band around 1581 cm^−1^ corresponding to the sp^2^ vibrational plane of the bonded carbon atoms in a 2D hexagonal lattice [31]. The I_D_/I_G_ ratio was calculated to determine the degree of disorder and the average size of sp^2^ domains in the prepared materials. The I_D_/I_G_ ratios of GO, ErGO, ErGO-cMWCNT, and ErGO-cMWCNT/AuNps were 1.04, 1.53, 1.21, and 1.22, respectively. The I_D_/I_G_ of GO confirms the presence of in-plane sp^2^ domains and sp^3^ hybridization due to the presence of oxygenated groups. The increased ratio in ErGO indicated the introduction of defects in the structure during the removal of oxygen functionalities after electrochemical reduction (Figure S1). I_D_/I_G_ values for ErGO-cMWCNT and ErGO-cMWCNT/AuNPs are lower than of ErGO, which may indicate that cMWCNT and AuNPs reduced the defect density by filling the spaces left after the remotion of oxygen functionalities.

### 3.2. Electrochemical Characterization

**Figure 4:**
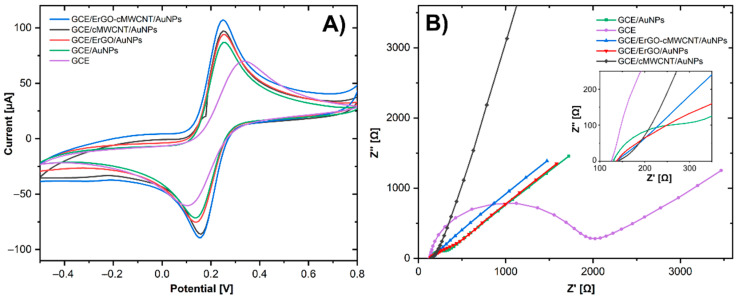
(**A**) CV in 5.0 mM [Fe(CN)_6_]^−3/−4^ containing PBS 0.01 M at 100 mV s^−1^, (**B**) EIS in 5.0 mM [Fe(CN)_6_]^−3/−4^ containing PBS 0.01 M.

Figure 4A shows the CVs of all CME and the bare GCE in [Fe(CN)_6_]^−3/−4^ as the redox probe to study the electrochemical behavior of the modified surfaces at a scan rate of 100 mV s^−1^. All the modifications showed well-defined redox peaks for the Fe(CN)_6_]^−3/−4^ redox couple. The values of the peak-to-peak separation (ΔE_p_) and the current intensity of the anodic peak (I_pa_) were also determined for GCE (ΔE_p_ = 219 mV, I_pa_ = 72 μA), GCE/AuNP (ΔE_p_ = 109 mV, Ipa = 90 μA), GCE/ErGOAuNP (ΔEp = 109 mV, Ipa = 94.42 μA), GCE/cMWCNT/AuNPs (ΔE_p_ = 89 mV, I_pa_ = 87 μA), and GCE/ErGO-cMWCNT/AuNPs (ΔE_p_ = 89 mV, I_pa_ = 101 μA). 

The bare GCE showed the largest ΔE_p_, which was significantly reduced with all the surface modifications, especially with ErGO-cMWCNT/AuNPs, which implies that the hybrid-based nanocomposite promotes a faster electronic transfer [32]. The results reveal great electrocatalytic features of GCE/ErGO-cMWCNT/AuNPs, due to the synergistic effect of AuNPs, ErGO, and cMWCNT, which improved the electron transfer rate, accelerating the oxidation/reduction response of the redox probe on the electrode surface [33]. 

The electrochemically active surface area of the various modified electrodes was evaluated using CV, varying the scan rates and using the [Fe(CN)_6_]^−3/−4^ solution (Figure S2). The surface area was calculated using the Randles–Sevick Equation (1):

*I_pa_ = 2.69 × 10^5^ A D½ n^3/2^ v½ C* (1)

where *n* is the number of electrons participating in the redox reaction (*n* = 1), *A* is the electroactive area (cm^2^), *v* is the scan rate (V s^−1^), and *D* and *C* are the diffusion coefficient (6.7 × 10^−6^ cm^2^ s^−1^) and the concentration (mol cm^−3^) of the redox probe, respectively [34]. The *A* was calculated using the slope of *I_pa_* versus the root of scan rate (*v*^1/2^). The resulting electroactive surfaces were GCE (0.055 cm^2^), GCE/AuNP (0.081 cm^2^), GCE/ErGO/AuNPs (0.088 cm^2^), GCE/cMWCNT/AuNPs (0.095 cm^2^), and GCE/ErGO-cMWCNT/AuNPs (0.11 cm^2^).

The results showed that GCE/ErGO-cMWCNT/AuNPs had the most prominent active electrochemical area among the CMEs, increasing the active surface of the GCE by 100%. The ErGO-cMWCNT/AuNP improved electrochemical response can be attributed to the generation of 3D networks formed by interconnected carbon nanotube–graphene hybrids and AuNPs, with well-defined nanopores [24,35]. The nanostructures led to a synergistic effect in enhanced conductivity, electroactivity, and enlarged electroactive surface area with multiple active sites, making it optimal for transduction in electrochemical sensing.

### 3.3. Electrochemical Detection of Dihydroxybenzene Isomers

EIS is an effective tool for studying the electrode–electrolyte interface properties of CMEs. In the Nyquist graph, the semicircular section at high frequency permits calculating the charge transfer resistance (R_ct_), while the linear part at low frequency is associated with the diffusion control process [15]. Figure 4B shows the EIS of the CMEs recorded in 5 mM of [Fe(CN)_6_]^−3/−4^, showing values of the R_ct_ of GCE (1788 Ω), GCE/AuNPs (135.28 Ω), GCE/ErGO/AuNPs (90 Ω), GCE/cMWCNT/AuNPs (26.97 Ω), GCE/ErGO-cMWCNT/AuNPs (24.2 Ω). The bare GCE has the largest R_ct_, but significantly decreased with all the modifications, especially with GCE/ErGO-cMWCNT/AuNPs, indicating that the incorporation of these materials improved the conductivity and led to faster charge transfer kinetics. These results evidenced the synergistic effect between ErGO, cMWCNT, and AuNPs, enabling a significant number of active sites for chemical reactions.

**Figure 5:**
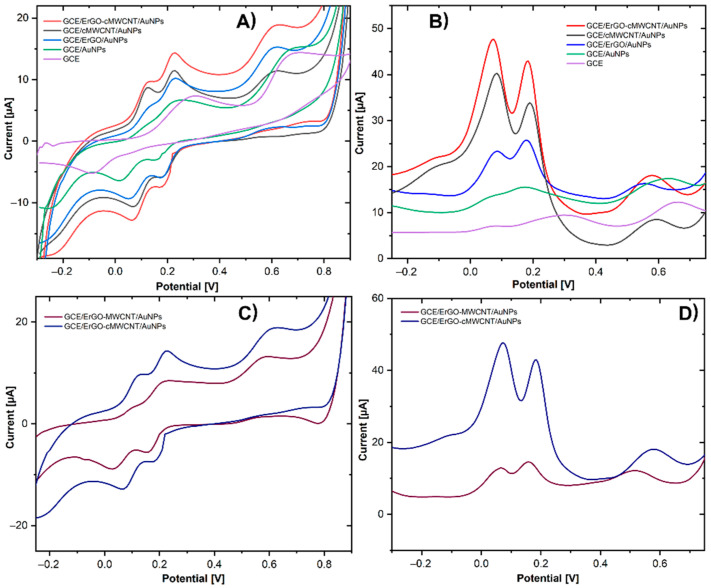
(**A**,**C**) Cyclic voltammetry of modified GCE electrodes in HQ, CC, and RS 0.2 mM in PBS 0.01 M at pH 7. (**B**,**D**) Differential pulse voltammetry of modified GCE electrodes in HQ, CC, and RS 0.2 mM in PBS 0.01 M at pH 7.

To study the electrochemical features of the DHB, the CME was tested in a PBS solution (0.01 M, pH 7) containing 0.2 mM of HQ, CC, and RS by CV and DPV. Figure 5A shows the CV of the electrodes. The overlapping redox peak of HQ and CC in GCE was attributed to the slow electron dynamics and limited diffusion of the isomers at the electrode surface [34]. The introduction of AuNPs and ErGO/AuNPs to the GCE induced a slight enhancement, which enabled the separation of the CC and HQ redox peak, showing three poorly defined oxidation peaks. In addition, the GCE modification with cMWCNT/AuNPs and ErGO-cMWCNT/AuNPs drastically improved the electrochemical performance, enabling the obtention of three well-separated oxidation peaks at 0.11 V, 0.22 V, and 0.6 V, attributed to the oxidation of HQ, CC, and RS. HQ and CC exhibited a reversible quinolinic form with a pair of redox peaks, while the oxidation reaction of RS is irreversible. This is attributed to the charge density, which is different in each isomer due to the position of the two hydroxyl functional groups on the benzene ring [33]. 

The electrochemical response of DHB at the different electrodes by DPV (Figure 5B) coincides appropriately with those measured by CV. It is clearly noticeable that the electrochemical activity of the GCE/ErGO-cMWCNT/AuNPs nanocomposite was superior for the detection of the three isomers than those at separate materials. The 3D network formed by the strong π–π stacking interaction between ErGO and cMWCNT, with well-defined nanopores and active catalytic sites of AuNPs, facilities the adsorption of the DHB and the electron transfer efficiency between the electroactive analytes and the CME. Additionally, to corroborate that carboxylation improves the electroactive performance of the pristine MWCNT, the nanocomposite was elaborated with either MWCNT or cMWCNT. The CME with the pristine MWCNT leads the separation of the CC and HQ oxidation peaks, but poorly defined (Figure 5C), whilst with cMWCNT (Figure 5D), showed separated and well-defined peaks for each DHB.

Moreover, the nanocomposite with the cMWCNT exhibited an increased current associated with the induction of the carboxylic groups to the MWCNT structure, facilitating the adsorption of organic molecules on the electrode surface. The π–π interaction between the phenyl structure of DHB and the hexagonal carbon structure and carboxyl groups of cMWCNT promotes the electron transfer [36]. It has been reported that the acid functionalization of MWCNT breaks the long nanotubes into shorter ones, increasing the number of edge plans, which provide a greater available area to the electroactive reactions, promoting a faster charge-transfer rate with better electrocatalytic activity [37,38]. Besides, the aggregation of graphene sheets was avoided not only by the π–π stacking interactions between the ErGO sheets and the walls of the cMWCNT but also by the steric barrier from the carboxylic groups of cMWCNT [39]. Moreover, the distribution of the cMWCNT on the graphene surface led to a larger anchoring surface area for AuNPs. Consequently, the highest electrocatalytic activity for the oxidation of HQ, CC, and RS was obtained with GCE/ErGO-cMWCNT/AuNPs nanocomposite, corroborating the synergistic effect of the components for enhancing electroactivity. 

### 3.4. Effect of pH and Scan Rate

**Figure 6:**
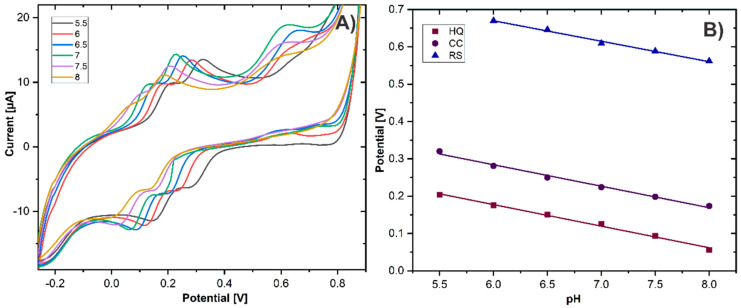
(**A**) Cyclic voltammetry of GCE/ErGO-cMWCNT/AuNPs for a mixture of 0.2 mM of HQ, CC, and RS in different pH solutions at 50 mV s^−1^. (**B**) Plots of effects of anodic peak potential vs. pH.

The electrochemical response of phenolic compounds is influenced by the pH because it affects the involvement of proton transference in the redox process [25]. Hence, the effect of pH on the electrochemical response of HQ, CC, and RS was evaluated by CV in the pH range of 5.5–8.0. Figure 6 shows the effects of the pH value on the electrochemical response of HQ, CC, and RS at the GCE/ErGO-cMWCNT/AuNPs. The potential of the oxidation peak E_pa_ at the DHB isomers shifted negatively as the pH increased, demonstrating that the protons were directly involved in the redox process [40]. The regression equations for three DHB isomers are as follows: HQ: E_pa_ (V) = −0.0578 pH + 0.5241 (R^2^ = 0.9929), CC: E_pa_ (V) = −0.0575 pH + 0.6295 (R^2^ = 0.9917), and RS: E_pa_ (V) = −0.0548pH + 0.999 (R^2^ = 0.9934).

The slopes of the three regression equations were −0.0578 V pH^−1^, −0.0575 V pH^−1^, and −0.0548 V pH^−1^ for HQ, CC, and RS, respectively. These values are very close to the theoretical value of −0.059 V pH^−1^ obtained from the Nernst equation.

**Figure 7:**
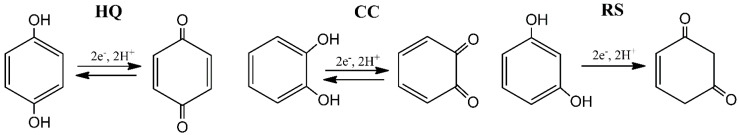
The electrooxidation mechanism of HQ, CC, and RS.

The results indicated that an equal number of protons and electrons took place in the redox reaction of HQ, CC, and RS [41], which is in agreement with previous works [33,34]. Based on this, the reaction mechanism of the DHB isomers is illustrated in Figure 7. Furthermore, within the pH range from 5.5 to 8.0, the maximum current intensity was achieved at pH 7. Thus, to obtain optimal sensitivity and selectivity, pH 7.0 was chosen for the simultaneous detection of HQ, CC, and RS.

**Figure 8:**
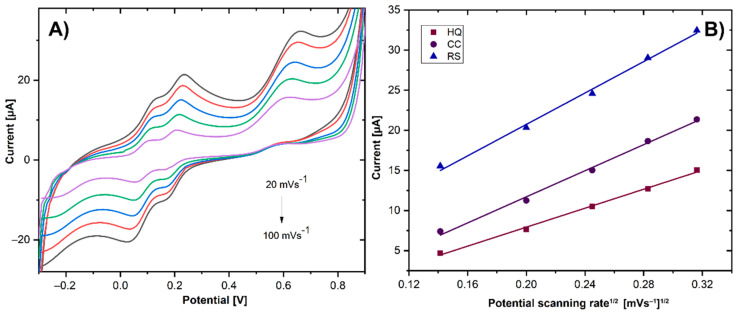
(**A**) Cyclic voltammetry of GCE/ErGO-cMWCNT/AuNPs in presence of HQ, CC, and RS at pH 7.0 with different scan rates. (**B**) Plots of effects of anodic peak potential vs. v^1/2^.

The electrooxidation process of DHB was studied by CV at scanning rates from 20 to 100 mV s^−1^ using 0.2 mM HQ, CC, and RS in 0.01M PBS, pH 7. As shown in Figure 8A, with the increasing scan rate, the redox peak currents of HQ, CC, and RS increased gradually. The linear relationship between the scan rate square and the anodic peak current (Figure 8B) confirmed that the electrooxidation process of the DHB at GCE/ErGO-cMWCNT/AuNPs is regulated by the diffusion-controlled electrochemical process [42]. The obtained regressions are expressed by HQ: I_pa_ (μA) = 59.128 v^1/2^ (V s^−1^)^1/2^ −3.8945 (R^2^ = 0.997), CC: I_pa_ (μA)= 81.139 v^1/2^ (V s^−1^)^1/2^ − 4.4874 (R^2^ = 0.9951), and RS: I_pa_ (μA) = 97.634 v^1/2^ (V s^−1^)^1/2^ + 1.2542 (R^2^ = 0.995).

Moreover, the potentials of the oxidation peaks of HQ, CC, and RS shifted to a more positive position, and the reduction peak potentials of HQ and CC shifted negatively with the increase in the scan rate. The shift of the peak potentials is associated with the size of the diffusion layer, which creates an internal resistance at the electrode–electrolyte interface [43]. Hence, the current is noticeably higher at the electrode surface when the potential is swept at higher scan rates [44].

According to Laviron’s theory [45], the charge-transfer coefficient (α) and the apparent heterogeneous electron transfer rate constant (K_s_) were calculated. The details of the equations are shown in the Supplementary Material. The α was calculated to be 0.574, 0.6103, and 0.41, and the values of k_s_ were 0.693 cm s^−1^, 0.973 cm s^−1^, and 0.49 s^−1^ for HQ, CC, and RS, respectively. These values show that the kinetics of charge-transfer are energetically favorable on the GCE/ErGO-cMWCNT/AuNPs surface.

### 3.5. Individual and Simultaneous Determination of HQ, CC, and RS

**Figure 9:**
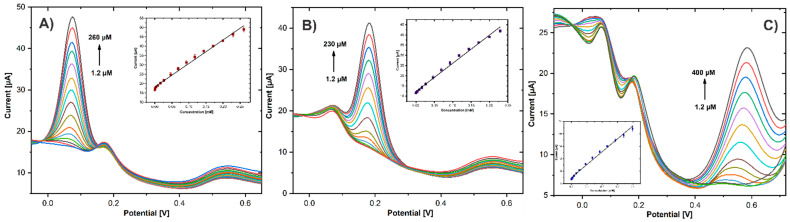
DPVs of GCE/ErGO-cMWCNT/AuNPs in 0.01 PBS (pH 7.0) containing (**A**) 25 μM CC, 50 μM RS, and different concentrations of HQ; (**B**) 25 μM HQ, 50 μM RS, and different concentrations of CC; (**C**) 25 μM HQ, 25 μM CC, and different concentrations of RS. Inset plots of the peak current as a function HQ, CC, and RS concentrations.

The quantitative analysis of HQ, CC, and RS on GCE/ErGO-cMWCNT/AuNPs was performed using DPV under optimized pH conditions. For single detection, the concentration of a given analyte was varied, and the other two were kept constant. Figure 9A shows the selective detection of HQ in the presence of 25 μM of CC and 50 μM of RS, with a typical response observed at 0.073 V. The peak current increased linearly with the HQ level in a range from 1.2 to 260 μM, while there was no evident increment in the response of the other analytes. Similarly, Figure 9B shows CC concentrations measured with 25 μM of HQ and 50 μM of RS in the solution. The peak current of CC was registered at 0.18 V, showing a linear response corresponding to CC concentrations without the interference of HQ and CC. The same behavior was observed for the current peak of RS at 0.58 V in the presence of 25 μM of HQ and 25 μM of CC, although a more comprehensive linear range was registered from 1.2 to 400 μM. The electrochemical reactions of the three DHB performed on the CME exhibited no influence on each other. The inset of Figure 9A–C exhibits the calibration curves of the anodic peak vs. the DHB concentration. The linear regression obtained for the single detection were HQ: I_pa_ (μA) = 124.57 C_HQ_ (μM) + 18.28 (R^2^ = 0.992), CC: I_pa_ (μA) = 137.02 C_CC_ (μM) + 12.39 (R^2^ = 0.993), and RS: I_pa_ (μA) = 41.27 C_RS_ (μM) + 6.0 (R^2^ = 0.992). The detection limits obtained for the single detection were 0.66, 0.84, and 0.39 μ M for HQ, CC, and RS, respectively.

**Figure 10:**
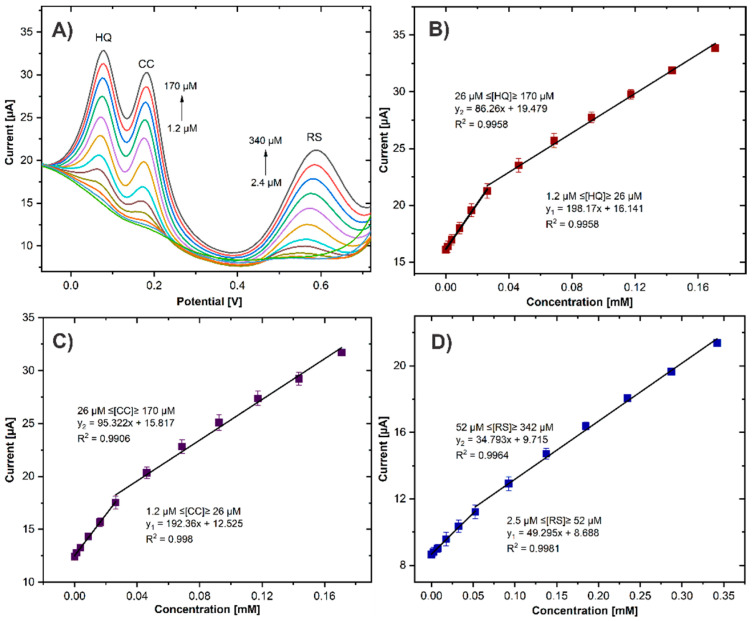
(**A**) DPVs of GCE/ErGO-cMWCNT/AuNPs in 0.01M PBS (pH 7.0) containing different concentrations of HQ, CC, and RS. (**B**–**D**) Plots of the oxidation peak currents as a function of HQ, CC, and RC concentrations, respectively.

The performance of the GCE/ErGO-cMWCNT/AuNPs sensor for multianalyte detection was evaluated using simultaneous increasing concentrations of HQ, CC, and RS. The oxidation currents linearly increased in proportion to analyte concentrations. Three well-defined and distinct oxidation peaks for HQ, CC, and RS are observed in Figure 10A. As shown in Figure 10B–D, the linear ranges of HQ, CC, and RS were divided into two, one at low concentrations and the other at high concentrations, as shown in Table 1, resulting in higher sensitivities for lower concentrations. The high sensitivity observed at lower concentrations is related to a more significant number of active sites on the electrode surface, which are progressively reduced as the concentration increases, consequently decreasing sensitivity. However, the calculated LOD (3.3SD/S) of 0.39, 0.54, and 0.61 μM for HQ, CC, and RS, respectively, showed the remarkable performance of the GCE/ErGO-cMWCNT/AuNPs sensor and the effective synergistic effects of the nanocomposite. This confirms that the CME can be used for simultaneous detection of the three DHB without interference among them.

Table 2 shows the comparison of the electrochemical parameters obtained in this work with devices designed for multianalyte detection of DHB. The comparative data showed that the electroanalytical properties of this work are comparable or even better concerning previously reported electrochemical sensors for simultaneous DHB detection. For instance, the GCE/ErGO-cMWCNT/AuNPs proposed in this work displays a more extensive linear range to HQ, CC, and RS and lower LOD to HQ and CC than those similar based on a 3D carbon nanotube–graphene hybrid decorated with gold nanoparticles [20]. In this work, the functionalization of MWCNT with carboxylic functional groups played an essential role in the good sensitivity towards DHB, because in addition to generating excellent dispersion in solution and enabling uniform electrode layer, it facilitated the adsorption of the DHB in both the electrode surface and the great amount of active edge plans with improved electrocatalytic activity. In addition, Table 2 displays that GCE/ErGO-cMWCNT/AuNPs had a notable performance with lower LOD and more extensive linear range than other similar carbon-based materials reported in the literature, such as graphene screen-printed electrode [46], Au-Pd nanoflower/reduced graphene oxide nanocomposite [47], and Cu-porphyrin-poly(styrene sulfonate) functionalized graphene [48]. The high performance of GCE/ErGO-cMWCNT/AuNPs expressed the notable synergistic effects granted to the nanocomposite.

### 3.6. Reproduciblity and Stability

**Figure 11:**
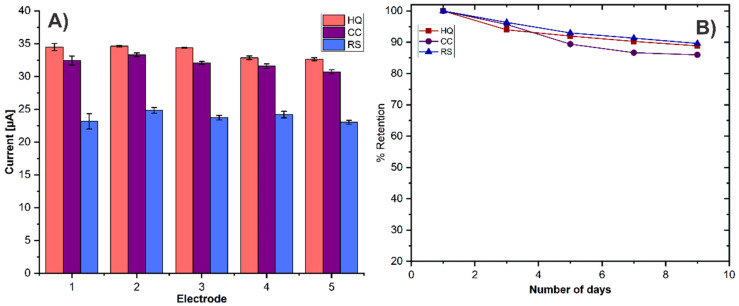
(**A**) Reproducibility of GCE/ErGO-cMWCNT/AuNPs of 5 different electrodes, (**B**) stability.

The reproducibility of the modified electrode GCE/ErGO-cMWCNT/AuNPs is crucial for the accurate monitoring of HQ, CC, and RS. Five electrodes were prepared by the same procedure and the electrochemical responses towards 0.2 mM of HQ, CC, and RS (Figure 11A) were compared. The results revealed that the relative standard deviations (RSD) of current peaks were 2.55%, 2.69%, and 2.81%, respectively. Thus, the electrode reproducibility was associated with better dispersion of the cMWCNT and the highly controlled electrochemical process for ErGO and AuNPs synthesis, which helps to generate a more homogeneous composite onto the electrode surface. 

The stability of the GCE/ErGO-cMWCNT/AuNPs sensor was also studied; the electrode was kept at room temperature (25 °C) in the air for nine days. During this period, the DPV detection was carried out under the same conditions every day. The stability was calculated by comparing the percentage (%) of current retention of the initial response. The % retention to HQ, CC, and RS reached 89%, 86%, and 90% of the initial value, respectively, manifesting high stability.

### 3.7. Real Sample Analysis

To demonstrate the applicability of the GCE/ErGO-cMWCNT/AuNPs sensor for the simultaneous quantitative detection of the DHB isomers, tap water and a skin-lightening commercial cream were tested. The results are summarized in Table 3. The concentrations of the DHB were calculated from the calibration plot, and the samples were evaluated by triplicate. Table 3 shows the recoveries of the samples in tap water, found in the range of 104.7–113.64% for HQ, 104.88–106.08% for CC, and 101.58–110.09% for RS. The obtained recoveries of the cosmetic sample were calculated to be 101.9–103.09% for HQ, 99.59–103.59% for CC, and 99.59–105.09% for RS. The satisfactory results demonstrate the feasibility of using GCE/ErGO-cMWCNT/AuNPs for the electrochemical detection of HQ, CC, and RS in environmental and cosmetic samples. Hence, the results confirmed that the proposed method has the potential for timely implementation and good reliability for the multiple detections of HQ, CC, and RS from different matrices. 

## 4. Conclusions

The GCE/ErGO-cMWCNT/AuNPs sensor presented a synergistic effect in enhanced conductivity, electroactivity, and enlarged electroactive surface area, associated with the 3D network formed by the strong π–π stacking interaction between ErGO and cMWCNT with well-defined nanopores, and the active catalytic sites of AuNPs. Additionally, the carboxylated MWCNT provided the nanocomposite with improved electrocatalytic properties towards the DHB detection compared to the pristine MWCNT. The GCE/ErGO-cMWCNT/AuNPs sensor allowed simultaneous detection of HQ, CC, and RS by monitoring their distinctive electrocatalytic oxidation current. The electrode provided acceptable linear ranges in the concentration range of 1.2–170 μM for HQ and CC and 2.4–400 μM for RS, as well as low detection limits for HQ, CC, and RS of 0.39 μM, 0.54 μM, and 0.61 μM, respectively. In addition, the electrochemical sensor showed good reproducibility, stability, and relevant results in the recovery rates of tap water and skin-lightening cosmetic cream. The results indicate that the proposed GCE/ErGO-cMWCNT/AuNPs electrochemical sensor has a great potential for the simultaneous, precise, and easy-to-handle detection of DHB in complex samples with high sensitivity.

## Figures and Tables

**Table 1:** Analytical parameters of GCE/ErGO-cMWCNT/AuNPs sensor for multianalyte detection.

Analyte	Potential (V)	Linear Range (μM)	Sensitivity (μA.mM.cm^−2^)	LOD (μM)
HQ	0.073	1.2–26, 26–170	1962, 854	0.39
CC	0.18	1.2–26, 26–170	1904, 922	0.55
RS	0.58	2.5–52, 52–342	488, 344	0.61

**Table 2:** Comparison of similar methods for the simultaneous determination of HQ, CC, and RS.

Modified Electrode	Real Sample	Linear Range (µM)			Detection Limit (µM)			References
		HQ	CC	RS	HQ	CC	RS	
3D CNT-Gr/AuNPs/GCE	Tap, river water	2–80	2–80	2–80	0.8	0.95	0.1	[20]
AuNPs/NfCAG/Gr-SPE	Tap, mineral water	0.2–75	0.2–50	0.2–125	0.014	0.017	0.05	[33]
ERGO-poly(PR)/AuNPs/GCE	Wastewater and cosmetic sample	0.1–90	0.4–90	4–350	0.053	0.053	0.079	[43]
Gr/SPE	Tap water	1–50	1–50	1–50	2.7	1.7	2.4	[46]
Au@PdNF/RGO/GCE	Tap, river, and lake water	1.6–100	2.5–100	2.0–100	0.5	0.8	0.7	[47]
PSS-Gr@Cu-TCPP/GCE	Lake, tap water	1–200	0.08–120	5–100	1	0.08	5	[48]
MWCNT@rGONR/GCE	Tap, river water	15–921	15–1101	15–1301	3.89	1.73	5.77	[49]
UiO-66/MC-3/GCE	Tap, lake water	0.5–100	0.4–100	30–400	0.056	0.072	3.51	[50]
GCE/ErGO-cMWCNT/AuNPs	Tap water, skin-lightening cream	1.2–170	1.2–170	2.5–342	0.39	0.54	0.61	This work

MWCNT, multiwalled carbon nanotube; rGONR, reduced graphene; NfCAG, Nafion-multi-walled carbon nanotubes aerogel; AuNPs, gold nanoparticles modified; Gr-SPE, graphene screen-printed electrode; NF, Nanoflower; rGO, reduced graphene oxide; Cu-porphyrin, Cu-TCPP; PSS-Gr, poly(styrene sulfonate) functionalized graphene; ERGO-poly(PR), electrochemically reduced graphene oxide-poly(Procion Red MX-5B); MOF-UiO-66, zirconium-based; MC, mesoporous carbon.

**Table 3:** Determination of HQ, CC, and RS in tap water and skin-lightening cream at the GCE/ErGO-cMWCNT/AuNPs (*n* = 3).

Scheme 3.	Included (μM)	Spiked (μM)			Found (μM)			Recovery (%)			% RSD (*n* = 3)		
Tap water		**HQ**	**CC**	**RS**	**HQ**	**CC**	**RS**	**HQ**	**CC**	**RS**	**HQ**	**CC**	**RS**
	-	0.05	0.05	0.1	0.056	0.052	0.105	113.64	105.40	101.58	8.97	3.06	4.75
	-	0.1	0.1	0.2	0.11	0.106	0.22	110.46	106.08	110.09	4.42	1.14	3.21
	-	0.15	0.15	0.3	0.157	0.157	0.321	104.70	104.88	107.18	8.54	3.46	5.22
Skin-lightening cream	0.111 HQ	-	0.11	0.22	0.114	0.110	0.253	103.09	100.50	105.09	1.92	6.12	1.09
	0.14 HQ	-	0.14	0.28	0.150	0.145	0.286	107.26	103.58	102.41	2.38	5.47	7.19
	0.17 HQ	-	0.17	0.34	0.173	0.169	0.344	101.79	99.59	101.179	4.12	0.95	0.47

## Data Availability

Not applicable.

## Supplementary Materials

The following are available online at https://www.mdpi.com/article/10.3390/bios11090321/s1, Figure S1: Raman spectra of GO and ErGO, Figure S2: Cyclic voltammetry of (a) GCE/AuNPs, (c) GCE/cMWCNT/AuNPs, (e) GCE/ErGO/AuNPs, (g) GCE/ErGO-cMWCNT/AuNPs at different scan rates in 5.0 mM [Fe(CN)_6_]^3−/4−^ containing PBS 0.01 M. The linear relationship between the anodic and cathodic peak currents versus root of the scan rate of (b) GCE/AuNPs, (d) GCE/cMWCNT/AuNPs, (f) GCE/ErGO/AuNPs, (h) GCE/ErGO-cMWCNT/AuNPs.
