# Supplementary material for: Simultaneous Detection of Dihydroxybenzene Isomers Using Electrochemically Reduced Graphene Oxide-Carboxylated Carbon Nanotubes/Gold Nanoparticles Nanocomposite
Source: Biosensors (Basel). 2021 Sep 7;11(9):321. doi: 10.3390/bios11090321 (PMC8468658; doi:10.3390/bios11090321)
Supplement: Supplementary file 1 [file biosensors-11-00321-s001.zip › biosensors-1347082-supplementary.pdf]

Supplementary material

Simultaneous Detection of Dihydroxybenzene Isomers Using Electrochemically Reduced Graphene Oxide-Carboxylated Carbon Nanotubes/Gold Nanoparticles Nanocomposite

Angélica Domínguez-Aragón [1], Rocio B. Dominguez [2] and Erasto Armando Zaragoza-Contreras [1,*]

[1] Centro de Investigación en Materiales Avanzados, S.C., Miguel de Cervantes No. 120, Chihuahua C.P. 31136, Chih, México; angelica.dominguez@cimav.edu.mx
[2] CONACyT-Centro de Investigación en Materiales Avanzados, S.C., Miguel de Cervantes 120, Chihuahua C.P. 31136, Chih, México; berenice.dominguez@cimav.edu.mx
* Correspondence: armando.zaragoza@cimav.edu.mx. Tel.: +52-614-439-4811; Fax: +52-614-439-1130

Citation: Domínguez-Aragón, A.; Dominguez, R.B.; Zaragoza-Contreras, E.A. Simultaneous Detection of Dihydroxybenzene Isomers Using Electrochemically Reduced Graphene Oxide-Carboxylated Carbon Nanotubes/Gold Nanoparticles Nanocomposite. *Biosensors* **2021**, *11*, 321. https://doi.org/10.3390/bios11090321

Received: 3 August 2021
Accepted: 2 September 2021
Published: 7 September 2021

Publisher's Note: MDPI stays neutral with regard to jurisdictional claims in published maps and institutional affiliations.

Copyright: © 2021 by the authors. Submitted for possible open access publication under the terms and conditions of the Creative Commons Attribution (CC BY) license (http://creativecommons.org/licenses/by/4.0/).

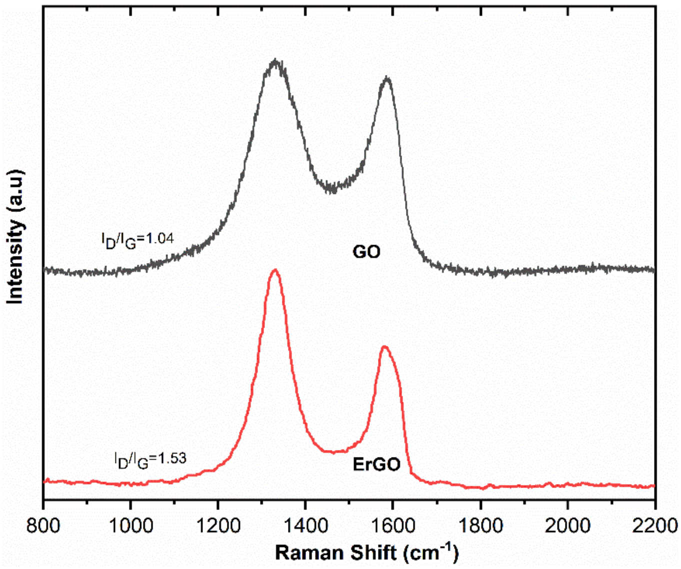

Figure S1. Raman spectra of GO and ErGO.

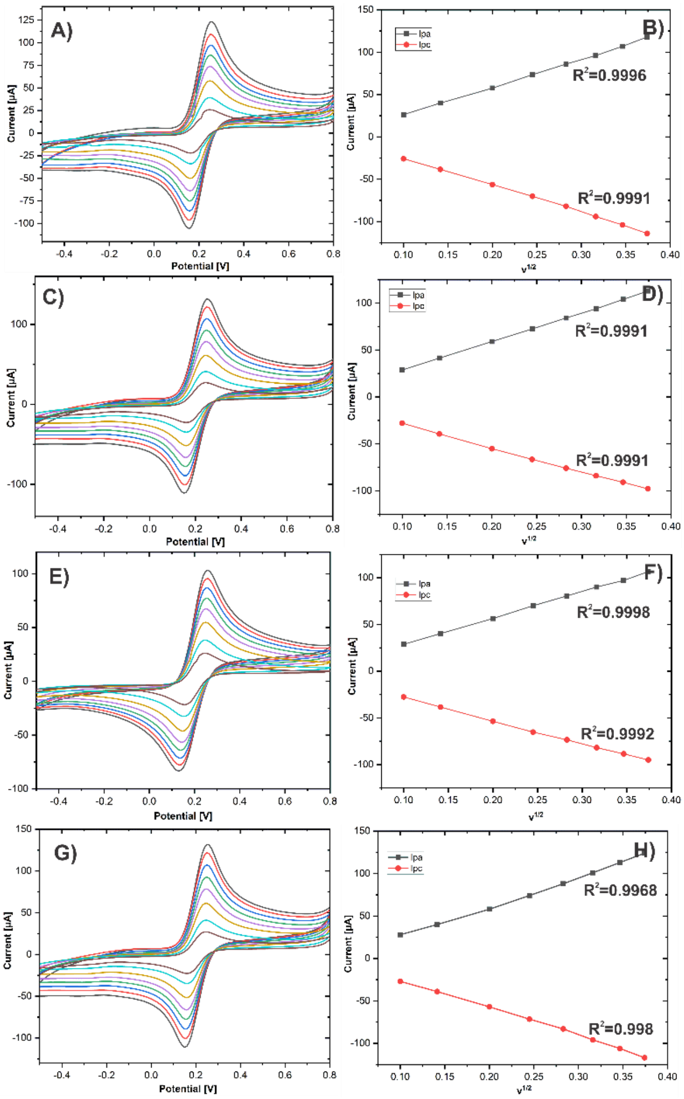

Figure S2. Cyclic voltammetry of A) GCE/AuNPs, C) GCE/cMWCNTAuNPs, E) GCE/ErGO-AuNPs, G) GCE/ErGOcMWCNTAuNPs at different scan rates in 5.0 mM [Fe(CN)6]$^{3-/4-}$ containing PBS 0.01 M. The linear relationship between the anodic and cathodic peak currents versus root of the scan rate of B) GCE/AuNPs, D) GCE/cMWCNTAuNPs, F) GCE/ErGOAuNPs, H) GCE/Er-GOcMWCNTAuNPs.

According to Laviron's theory [1] a reversible electrochemical process produces two straight lines with a slope equal to $-2.3RT/\alpha nF$ for the cathodic peak and $2.3RT/(1-\alpha)nF$ for the anodic peak. The charge transfer coefficient, α, can be calculated based on the slopes of the two straight lines of E_p *vs* log v using the following Equation (2):

$$\frac{k_a}{k_c} = \frac{\alpha}{1-\alpha} \qquad (2)$$

Where k_a and k_c are the slope of the straight lines for E_{pa} *vs* log v and E_{pc} *vs* log v, respectively. For HQ and CC (reversible process), the charge transfer coefficient (α) was calculated. The E_{pa} and E_{pc} are linearly dependent on the log v, represented by the followed equations: E_{pa} (HQ) = 0.0376 log v + 0.1582 (R^2 = 0.9909), E_{pc} (HQ) = -0.0278 log v + 0.0112 (R^2 = 0.9918), E_{pa} (CC) = 0.0412 log v + 0.2734 (R^2 = 0.9962), E_{pc} (CC) = -0.0263 log v + 0.1457 (R^2 = 0.995). Thus, the α was calculated to be 0.574 for HQ and 0.6103 for CC. In

addition, the apparent heterogeneous electron transfer rate constant (K_s) was also calculated using the follow Equation (3):

$$\log ks = \alpha\log(1-\alpha) + (1-\alpha)log\alpha - log\frac{RT}{nFv} - \frac{\alpha(1-\alpha)nF\Delta Ep}{2.3RT} \quad (3)$$

where n is the number of electrons involved in the reaction, the ΔE_p is the peak-to-peak separation, α is the charge transfer coefficient, v is the scan rate, R is the gas constant (8.314 J K−1 mol^{-1}), T is the temperature (298 K), and F is the Faraday constant (96,485 C mol^{-1}). The number of electrons involved in the reaction of HQ and CC was 2. Therefore, the values of k_s were calculated to be 0.693 cm s^{-1} and 0.973 cm s^{-1} for HQ and CC, respectively. These results indicated that the GCE/ErGO-cMWCNT/AuNPs promotes electron transfer effectively.

Due to the irreversible oxidation reaction of RS, it was used the Laviron's Equation (4) for the irreversible electrode process to calculate αn and k_s values [2].

$$Epa = E_0 - \left(\frac{RT}{\alpha nF}\right)\ln\left(\frac{RTk_s}{\alpha nF}\right) + \left(\frac{RT}{\alpha nF}\right)\ln v \quad (4)$$

Where α is the charge transfer coefficient, n is the transfer electron number, k_s is the heterogeneous electron transfer rate constant, R, T, and F have their usual meanings. The αn was calculated as 0.82 and k_s value of RS was 0.49 s^{-1} based on the slope and intercept of E_{pa} vs ln ν equation, respectively. Because the electron number involved in the oxidation process is 2, α was calculated to be 0.41.

References

1. Laviron, E. General expression of the linear potential sweep voltammogram in the case of diffusionless electrochemical systems. J. Electroanal. Chem. 1979, 101, 19–28, doi:10.1016/S0022-0728(79)80075-3.
2. Zhang, C.; Han, M.; Yu, L.; Qu, L.; Li, Z. Fabrication an electrochemical sensor based on composite of Cu-TCPP nanosheets and PSS functionalized graphene for simultaneous and sensitive determination of dihydroxybenzene isomers. J. Electroanal. Chem. 2021, 890, 115232, doi:10.1016/j.jelechem.2021.115232.
